# Crystallization and Quantification of Crystalline and Non-Crystalline Phases in Kaolin-Based Cordierites

**DOI:** 10.3390/ma12193104

**Published:** 2019-09-23

**Authors:** Marta Valášková, Zdeněk Klika, Boris Novosad, Bedřich Smetana

**Affiliations:** 1 Institute of Environmental Technology, VŠB-Technical University of Ostrava, 17. listopadu 15, 708 00 Ostrava-Poruba, Czech Republic; 2 Department of Chemistry, VŠB-Technical University of Ostrava, 17. listopadu 15, 708 00 Ostrava-Poruba, Czech Republic; zdenek.klika@vsb.cz; 3 Nanotechnology Centre, VŠB-Technical University of Ostrava, 17. listopadu 15, 708 00 Ostrava-Poruba, Czech Republic; boris.novosad.st@vsb.cz; 4 Faculty of Electrical Engineering and Computer Science, VŠB-Technical University of Ostrava, 17. listopadu 15, 708 00 Ostrava-Poruba, Czech Republic; 5 Faculty of Materials Science and Technology, VŠB-Technical University of Ostrava, 17. listopadu 15, 708 00 Ostrava-Poruba, Czech Republic; bedrich.smetana@vsb.cz

**Keywords:** raw kaolins, cordierite/indialite, thermal conversions, X-ray diffraction, chemical quantitative mineral analysis

## Abstract

Kaolin is most often used as traditional raw material in ceramic industry. The purpose of the study was to obtain understanding of the structural and chemical variability of cordierite ceramics influenced by chemical and mineralogical properties of six raw kaolins taken from different localities when they are applied in ceramics mixtures with vermiculite and sintered up to 1300 °C. The X-ray diffraction and simultaneous thermogravimetric and differential thermal analysis were used to identify and characterize crystalline mineral phases and the course of reactions during the heating. The percentages of the crystalline and non-crystalline phases were newly determined by recalculation of the bulk chemical analyses of kaolins and cordierite ceramics using Chemical Quantitative Mineral Analysis (CQMA) method. Varying amounts of minerals in kaolins: kaolinite from 73.3 to 85.0, muscovite from 4.2 to 9.9, and quartz from 6.0 to 19.5 (mass %) affected amount of cordierite/indialite from 75.2 to 85.1, enstatite from 5.8 to 8.9 (when are calculated as their maximal possible percentages), and non-crystalline phases from 8.8 to 15.1 (mass %) in cordierite ceramics. Regression analysis predicted high relationship between quantity of: (a) kaolinite in kaolins and crystalline cordierite and (b) quartz in kaolins and non-crystalline phases in the ceramics. The migration of potassium from muscovite into the cordierite structure, melting point and crystallization of cordierite/indialite phases and pore size variability in relation to impurity of kaolins are documented and discussed.

## 1. Introduction

Cordierite (Mg_2_Al_4_Si_5_O_18_) has the oxide ratio composition 2:2:5 of 2MgO·2Al_2_O_3_·5SiO_2_, and is one of the phases, which are present in the ternary glass–ceramic system MgO–Al_2_O_3_–SiO_2_ [1]. Cordierite-like crystals synthesized below 830 °C were termed β form [2]. The µ-cordierite was considered as a metastable product crystallizing from glass at temperatures below 950 °C which transforms irreversibly upon heating to the α form. The low temperature orthorhombic form is cordierite stable below 1450 °C. The high temperature hexagonal form is disordered indialite stable above 1450 °C [3], which below 1450 °C transforms slowly to β phase [4]. The conventional methods for the synthesis of cordierite ceramics include solid-state sintering of magnesium, aluminum, and silicon oxides in the mixtures corresponding to chemical composition of cordierite. Addition of nucleating agents (e.g., TiO_2_, ZrO_2_, Fe_2_O_3_) effectively increases the softening and decreases glass transition temperatures.

The oxide ratio in composition of cordierite is economically produced from the raw kaolin containing kaolinite (Al_2_O_3_∙2SiO_2_∙2H_2_O), talc (3MgO∙4SiO_2_∙H_2_O) and a correcting component alumina (Al_2_O_3_). The fact that the properties of clay minerals used in the mixture and the conditions of preparation influence sintering and properties of ceramic body is widely known. Structural properties of cordierites prepared by the solid-state reaction from the mixtures of talc, kaolin, and alumina as well as from kaolin and vermiculite without further correcting additives at 1300 °C were characterized in the previous works [5,6]. Raw kaolins contain kaolinite, muscovite (white mica), quartz, and an admixture of silicates, mostly feldspars, biotite, and accessory minerals [7]. Transformation of kaolin containing kaolinite, quartz, and muscovite in the temperature range of 980–1121 °C produced primary mullite with a Si-Al spinel [8,9]. Impurities in kaolins such as muscovite and K-feldspar, containing potassium in their structure, act as flux that facilitated easy melting upon firing and formation of non-crystalline glassy phase [10,11,12]. Similarly, Fe-impurities supported crystallization of mullite [13]. Interaction between kaolinite and muscovite in the temperature range of dehydroxylation of muscovite between 800–900 °C was not declared [14]. The minerals of the mica groups transformed at 950 °C to mullite, K-feldspar and plagioclase [15]. At temperatures below 1000 °C, muscovite was replaced by alkali feldspars and quartz was preserved even at 1100 °C, while cristobalite was identified only in quartz-rich clays [11].

The main aim of this work was to obtain an understanding of the structural and chemical variability of cordierite ceramics influenced by chemical and mineralogical properties of kaolins taken from different localities. Therefore, the simple two-phase ceramic mixtures of kaolin and vermiculite without correcting additives were prepared in the mass ratio 1:1, close to the oxide ratio composition 2:2:5 of cordierite 2MgO·2Al_2_O_3_·5SiO_2_ for the solid state reaction up to 1300 °C. X-ray diffraction and simultaneous thermogravimetric and differential thermal analysis were used to characterize crystalline mineral phases and decomposition and crystallization of phases from the melt. Quantification of crystalline and non-crystalline phases is newly performed based on the elemental bulk chemical analysis using the Chemical Quantitative Mineral Analysis (CQMA) method [16]. 

## 2. Material and Methods

### 2.1. Kaolin Samples

The granite-derived kaolins of the Karlovy Vary region and kaolins from K-feldspar-rich sedimentary rocks of the Pilsen region represent two main Czech kaolin deposits [17,18]. The three kaolin samples originating from the Karlovy Vary region are from personal collection and are labeled as Bo (locality Božičany), Se (locality Sedlec) and Po (locality Podbořany). Kaolin samples originating from the Pilsen Basin were obtained from LB Minerals, Ltd., Czech Republic, and are labelled as Ka (KKN from locality Kaznějov), Br (locality Horní Bříza) and Ch (DSF from locality Chlumčany). All kaolin samples were subjected to sieving through 0.04 mm sieve.

### 2.2. Kaolin–Vermiculite Ceramic Mixtures

Vermiculite from the Paraiba region of Brazil (Ver) [19] was supplied by Grena, Ltd., Veselí nad Lužnicí, Czech Republic. Sample (30 g) was milled using agate planetary mill Fritsch Pulverisette 5 and then sieved to the fraction ˂40 µm. 

The ceramic kaolin–vermiculite mixtures marked as C-Bo, C-Se, C-Po, C-Ka, C-Br, and C-Ch were prepared in their mass ratio of 1:1, homogenized for 1 h (Heidolph Reax overhead shaker, REAX 20/4, Merck, Darmstadt, Germany), and then milled for 15 min at 550 rpm in agate planetary mill Fritsch Pulverisette 5. Samples were prepared in a porcelain combustion boat and then sintered in an electrical laboratory furnace LH15/13 at the heating rates 10 °C min^−1^ up to 1000 °C and 2 °C min^−1^ to 1300 °C. The samples were then left at this temperature for 1 h and after that slowly cooled to the room temperature. The ceramic samples were marked identically as their parent ceramic mixtures. 

### 2.3. Methods 

Elemental analysis was performed using the SPECTRO XEPOS energy dispersive X-ray fluorescence (ED-XRF) spectrometer (Spectro Analytical Instruments, Kleve, Germany). The results from the XRF analysis were calculated to the stoichiometric metal oxides concentrations. 

Simultaneous thermogravimetric (TG) and differential thermal analysis (DTA) of samples was performed in platinum crucibles using the experimental system SETARAM SETSYS 18 TM TG/DTA/TMA (Lyon, France) with an “S”-type measuring rod (tricouple) in argon atmosphere (6N) at a heating rate of 10 °C min^−1^ from 25 to 1300 °C. The transition temperatures determined according DTA with an estimated error of ±5 °C. 

The X-ray diffraction (XRD) analysis of crystalline mineral phases was carried out on the X-ray diffractometer Ultima IV (RIGAKU, Tokyo, Japan). The XRD patterns were recorded in the 8–60° 2θ range (CuKα radiation λ = 0.15418 nm, a scintillation detector and a scanning rate of 2 °/min at 40 kV and 40 mA). The porosity of the dried ceramic samples was measured using a mercury intrusion porosimeter AutoPore IV 9500 (Micromeritics Instrument Corporation, Norcross, GA, USA). All methods listed here are more fully described in the previous work [6,20].

The Chemical Quantitative Mineral Analysis (CQMA) method recalculates the elemental bulk chemical analysis (analytes SiO_2_, Al_2_O_3_, etc.) to the identified minerals in XRD analysis and their crystallochemical formulas of the analyzed sample. The calculation of mineral amounts is performed by optimization procedures (e.g., the least square and/or non-negative least square method). The additional more detailed information as well as recalculation of the quantitative content of minerals determined by other methods to their elemental bulk chemical analysis has recently been published [16]. 

## 3. Results

### 3.1. Kaolin Samples 

**Figure 1:**
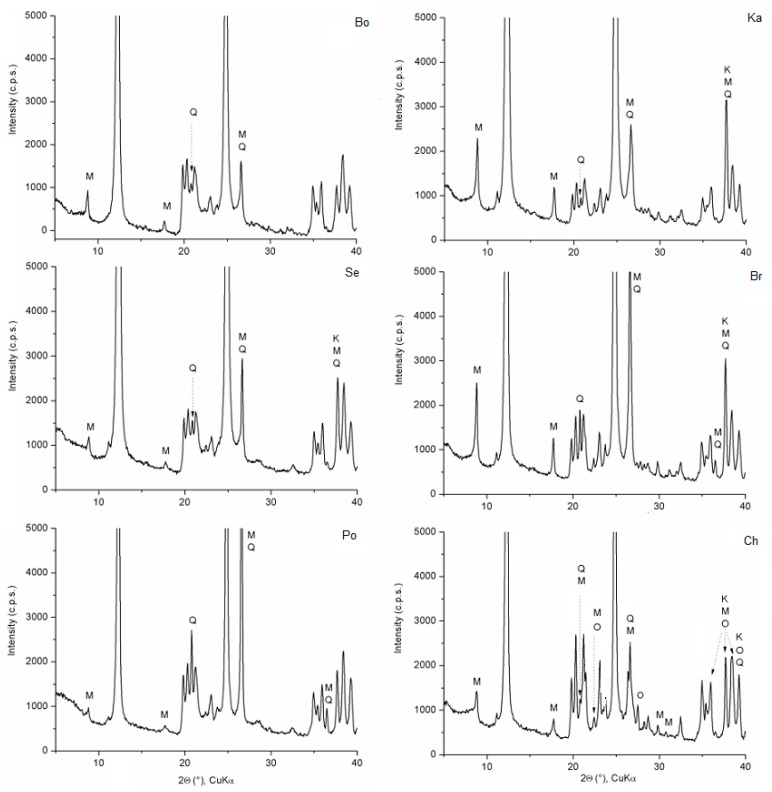
XRD patterns of kaolin samples from 5 to 40° 2θ. Peaks of kaolinite (K) are unlabeled; M—muscovite, Q—quartz, and O—orthoclase.

The chemical compositions of kaolin samples indicate not pure kaolinites (Al_2_O_3_·2SiO_2_·2H_2_O) (Table 1). The XRD patterns of kaolins (Figure 1) showed kaolinite (PDF card no. 00-058-2005), muscovite (PDF card no. 01-076-0668), quartz (PDF card no. 01-086-2237), and orthoclase (PDF card no. 01-075-1592) in the kaolin sample Ch. 

**Figure 2:**
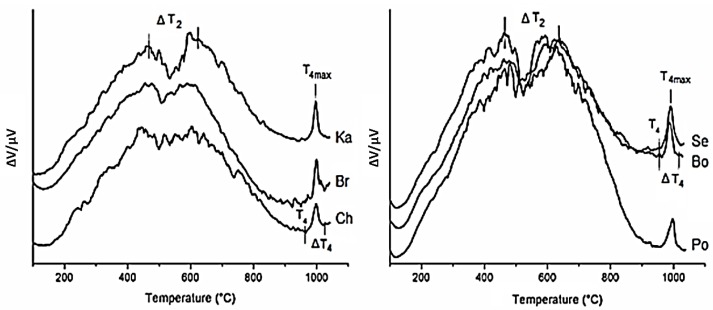
DTA curves of kaolin samples.

The DTA/TG analysis (DTA curves in Figure 2) of kaolins heated to 1100 °C displayed thermal events occurring within the temperature intervals characteristic for transformation of kaolinite and micas (Table 2).

The temperature conversions of kaolins containing admixture of muscovite and quartz produce new phases that will be involved in the sintering and crystallization process of cordierite-indialite.

The temperature range ΔT_1_ = 20–200 °C correspond to the loss of adsorbed water [22,23]. The temperature range ΔT_2_ = 400–730 °C was assigned to dehydroxylation of kaolinite and conversion to metakaolinite (e.g., [11,22,24]), according to the reaction (Equation (1))

(1) $$\underset{\mathrm{Kaolinite}}{{\mathrm{Al}}_{2}{\mathrm{Si}}_{2}O_{5}{\left(\mathrm{OH}\right)}_{4}}\;\overset{400–730\;{}^{\circ}C}{\to }\;\underset{\mathrm{Metakaolinite}}{{\mathrm{Al}}_{2}{\mathrm{Si}}_{2}O_{7}\;+\;2H_{2}O}$$

The position of the endothermic minimum T_2min_ moving from 526 °C to 535 °C was consistent with structural ordering, expressed by Hinckley index [20,25,26] (Table 1).

The temperature range ΔT_3_ = 720–946 °C of an endothermal process accompanied by the mass loss Δm_3_ about 0.67 ± 0.17% was assigned to dehydroxylation of muscovite [11,27]. The transformation temperature from muscovite into dehydroxylated muscovite were reported in the various ranges (e.g., 300–1000 °C [27], 650–750 °C [28], 850–1000 °C [29]), according to the reaction (Equation (2))

(2) $$\underset{\mathrm{Muscovite}}{{\mathrm{KAl}}_{2}({\mathrm{AlSi}}_{3})O_{10}{\left(\mathrm{OH}\right)}_{2}}\;\overset{900\;{}^{\circ}C}{\to }\;\underset{\mathrm{Dehydroxylated muscovite}}{{\mathrm{KAl}}_{2}({\mathrm{AlSi}}_{3})O_{11}}\;+\;H_{2}O$$

The temperature range ΔT_4_ = 955 °C–1051 °C and the exothermic maximum from 988–999 °C (marked as T_4max_ in Table 2) was assigned to the transformation of metakaolinite to primary mullite. Other small diffuse exothermic effects above 1050 °C were attributed to the secondary mullite [29]. The processes of the formation of primary and secondary mullites from the kaolin refractory clays were mostly described by the reactions (Equations (3)–(5)) [12,29,30]

(3) $$\underset{\mathrm{Metakaolinite}}{{\mathrm{Al}}_{2}{\mathrm{Si}}_{2}O_{7}}\;\overset{550–1200\;{}^{\circ}C}{\to }\;\underset{\mathrm{Primary mullite}}{\left(3{\mathrm{Al}}_{2}O_{3}\cdot 2{\mathrm{SiO}}_{2}\right)}\;+\;{\mathrm{SiO}}_{2}\;(\mathrm{amorphous})$$

Depending on the structural defects of kaolinites, metakaolinite structure decomposes at about 900 °C with segregation of alumina and silica according to the reaction (Equation (4)) [31]

(4) $$\underset{\mathrm{Metakaolinite}}{{\mathrm{Al}}_{2}{\mathrm{Si}}_{2}O_{7}}\overset{900\;{}^{\circ}C}{\to }\;\underset{\mathrm{Alumina}}{\gamma -{\mathrm{Al}}_{2}O_{3}}\;+\;2{\mathrm{SiO}}_{2}$$

A very diffuse DTA exothermic peaks observed in the region of 1100–1200 °C was attributed to a recombination reaction of the segregated phases (Equation (4)) and then to the formation of secondary mullite (Equation (5))

(5) $$4{\mathrm{SiO}}_{2}\;+\;6\gamma -{\mathrm{Al}}_{2}O_{3}\;\overset{>1050\;{}^{\circ}C}{\to }\;\underset{\mathrm{Secondary mullite}}{2(3{\mathrm{Al}}_{2}O_{3}\cdot 2{\mathrm{SiO}}_{2})}$$

Structure of dehydroxylated muscovite is unstable and easily melts in the temperature of the formation stage of mullite (Equation (6)). The initial stage of mullite crystals in SiO_2_—melt can be supported by the presence of K_2_O released upon melting from the dehydroxylated muscovite [32] according to the reaction (Equation (6))

(6) $$\underset{\mathrm{Dehydroxylated muscovite}}{2{\mathrm{KAl}}_{2}({\mathrm{AlSi}}_{3})O_{11}}\overset{1125–1150\;{}^{\circ}C}{\to }\;\underset{\mathrm{Mullite}}{\left(3{\mathrm{Al}}_{2}O_{3}\cdot 2{\mathrm{SiO}}_{2}\right)}\;+\;K_{2}O\;+\;4{\mathrm{SiO}}_{2}$$

### 3.2. Cordierite Samples 

The elemental composition of the ceramic samples prepared at 1300 °C was recalculated to the stoichiometric oxides. Fe was recalculated to Fe_2_O_3_ (Table 3).

**Figure 3:**
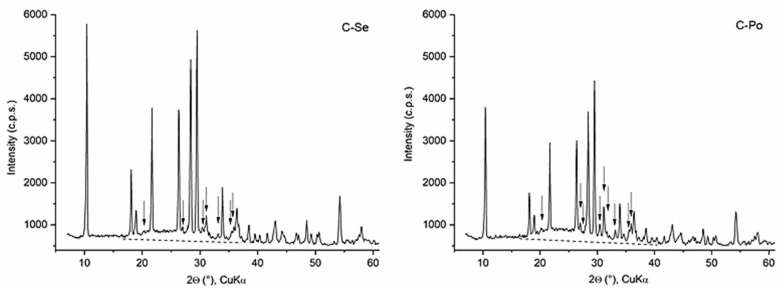
XRD patterns of ceramic samples C-Se and C-Po. The peaks of indialite are unmarked and enstatite is marked with arrow. A non-crystalline phase area is separated from the background by a dashed line.

**Figure 4:**
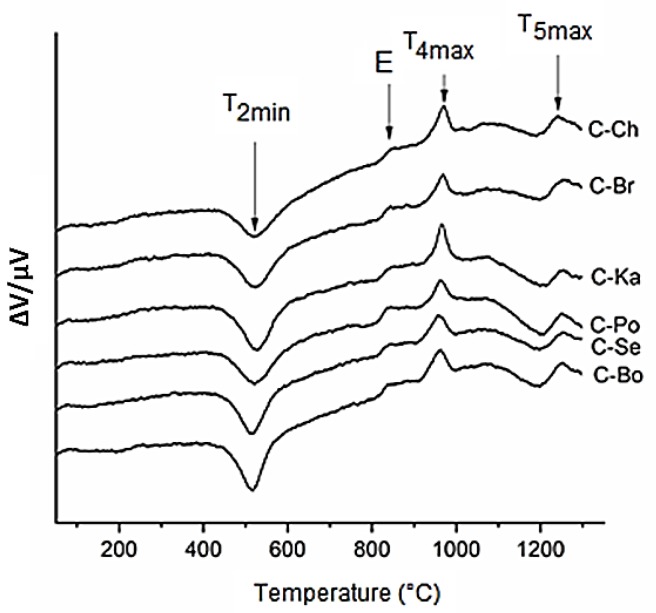
DTA curves of kaolin–vermiculite mixtures. E—enstatite.

The XRD patterns of ceramic samples were very similar and two ceramic samples C-Se and C-Po are given as examples (Figure 3). The crystalline indialite (PDF card No. 01-082-1884), enstatite (PDF card no. 00-019-0768) and a broad area of non-crystalline phases were identified at all ceramic samples. The TG/DTA results of kaolin–vermiculite mixtures (Table 4) and DTA curves (Figure 4) were evaluated as follows: dehydration of vermiculite and kaolinite have occurred in the temperature range ΔT_1_ = 20–265 °C. The temperature range of ΔT_2_ = 407–437 °C, accompanied by a mass loss of approximately 6.58 ± 0.30%, was assigned to dehydroxylation of kaolinite and a continuing dehydration and dehydroxylation of vermiculite. Vermiculite dehydroxylated and crystallized to enstatite at about 835 °C at the temperature range ΔT_3_ = 745–848 °C (marked E in Figure 4) [33,34]. The exothermal reactions in the range ΔT_4_ = 905–1017 °C and maximum peaks at T_4max_ = 960–972 °C have been attributed to the formation of MgAl_2_O_4_ spinel and μ-cordierite [35,36]. The temperature range ΔT_5_ corresponds to the melting and crystallization of indialite and enstatite. 

The transformation temperatures of the mineral phases and the temperatures prior to the crystallization process during the sintering of the kaolin–vermiculite ceramic mixtures on the TG-DTA curves (Figure 4) were determined at the five ranges (Table 4).

## 4. Discussion

The process of the formation of cordierite/indialite from kaolin–vermiculite mixtures can be generally described by the reactions including transformation of vermiculite to enstatite (Equation (7))

(7) $$\underset{\mathrm{Vermiculite}}{2\;{\mathrm{Mg}}_{3}({\mathrm{Si}}_{3}{\mathrm{Al}}_{1})O_{10}{\left(\mathrm{OH}\right)}_{2}}\;\overset{700–900\;{}^{\circ}C}{\to }\;\underset{\mathrm{Enstatite}}{6{\mathrm{MgSiO}}_{3}}\;+\;{\mathrm{Al}}_{2}O_{3}\;+\;H_{2}O$$

and then, the reaction between enstatite, mullite and SiO_2_ into cordierite/indialite formation (Equation (8))

(8) $$\underset{\mathrm{Enstatite}}{6{\mathrm{MgSiO}}_{3}}\;+\;\underset{\mathrm{Mullite}}{2(3{\mathrm{Al}}_{2}O_{3}\cdot 2{\mathrm{SiO}}_{2})}\;+\;5{\mathrm{SiO}}_{2}\;\overset{1250\;{}^{\circ}C}{\to }\;\underset{\mathrm{Cordierite/Indialite}}{3{\mathrm{Mg}}_{2}{\mathrm{Al}}_{4}{\mathrm{Si}}_{5}O_{18}}$$

The previous literature has confirmed the facts, that the impurity products react with the liquid phase influencing crystallization. Therefore, the position of the exothermic peak shifts to the lower temperatures, e.g., the presence of 1.5 mass % K_2_O lowered temperature about 25 °C [37]. 

The CQMA procedure [16] calculated from the bulk chemical analyses crystallochemical formulas and quantity of minerals, which were identified using the XRD patterns (Figure 1). In the case of kaolin samples, the CQMA recalculated all elements (Table 1) to the kaolinite Al_2_Si_2_O_5_(OH)_4,_ quartz SiO_2_, orthoclase KAlSi_3_O_8_ and muscovite, whose crystallochemical formula has been refined to K_0.87_Na_0.07_(Al_1.43_Fe^3+^_0.07_Mg_0.50_Ti_0.02_)(Si_3.39_Al_0.61_)O_10_(OH)_2_, and residual elements Fe and Ti to the commonly occurring minor phases in kaolins as limonite Fe_2_O_3_∙H_2_O and rutile TiO_2_ (Table 5).

The results in the Table 5 revealed similar amounts of kaolinites in kaolin samples Bo, Se, and Po from the Karlovy Vary region (80.1 ± 6.1 mass %) and kaolin samples Ka, Br, and Ch from the Pilsen area (80.4 ± 1.8 mass %), except the kaolin sample Po, which is poor in kaolinite (73.3 mass %) at the expense of quartz (19.5 mass %). All kaolins contain K-bearing muscovite in amounts lower than 10 mass % and orthoclase (8 mass % in kaolin sample Ch). 

In the case of ceramic samples, the CQMA recalculated all elements of the bulk chemical analyses (Table 3) to the quantitative amounts of crystalline minerals indialite/cordierite and enstatite (MgSiO_3_), identified by XRD patterns (Figure 2). The refined crystallochemical formula Ca_0.1_Mg_1.90_Fe^3+^_0.40_Al_3.60_Si_5.00_O_18_ of cordierite/indialite includes all content of Fe and Ca determined at the six ED-XRF analyses of ceramic samples (Table 3). It should be noted that a similar crystallochemical formula of indialite was reported by Balassone et al. [38]. The excess Si and all percentages of Ti, Na, and K were calculated as the oxidic non-crystalline phases based on 10SiO_2_ (Table 6), while considering all elements—Al, Fe, Mg, and Ca (Table 3)—already included in crystallochemical formulas of crystalline cordierite/indialite and enstatite. 

**Figure 5:**
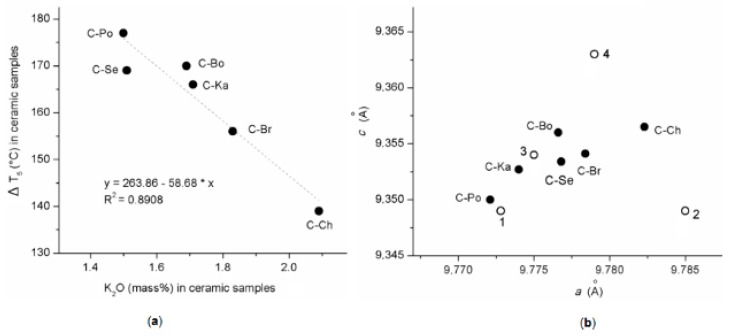
Relationsships between: (**a**) temperature ΔT_5_ vs. amount of K_2_O in ceramic samples and (**b**) unit cell parameters *c* vs. *a* of indialites. The parameters labeled as: 1: Mg_2.00_Al_4.00_Si_5.00_O_18_ [4]; 2: Mg_2.00_Al_4.00_Si_5.00_O_18_ [44]; 3: (K_0.17_Mg_1.94_Fe_0.06_Ca_0.04_)Al_4.25_Si_4.75_O_18_ [38]; and 4: (K_0.25_Mg_1.75_)Al_4.25_Si_4.75_O_18_ [43].

The quantitative amounts of minerals in ceramic samples determined using CQMA method (Table 6) can then be considered as the calculated percentages of the highest possible crystalline phases and the lowest possible non-crystalline phases. In six cordierite samples, the amount of cordierite/indialite varied from 75.2 to 85.1 mass %, enstatite from 5.8 to 8.9 mass % and non-crystalline phases from 8.8 to 15.1 mass %, depending on the purity of kaolins, i.e., containing kaolinite from 73.3 to 85.0, muscovite from 4.2 to 9.9 and quartz from 6.0 to 19.5 (mass %). The temperature corresponding to the melting and crystallization of indialite at the stage ΔT_5_ varied from 177 to 139 °C (Table 4) in the relation to the amount of K_2_O from 1.50 mass % to 2.09 mass % (Table 3). The relation in Figure 5a between the temperature ΔT_5_ (Table 4) and the amount of K_2_O in the ceramic mixtures (Table 3) was described by the linear regression function (Equation (9))

ΔT_5_(°C) = 263.86 − 58.68 × K_2_O, the correlation coefficient R^2^ = 0.891 (9)

The relationship makes it possible to determine the maximum K_2_O difference of 0.59% by mass between C-Po and C-Ch and corresponding to the ΔT_5_ temperature reduced by 42 °C. 

A high cordierite, generally prepared by ceramic procedures at 1300 °C, crystallizes in a pseudohexagonal symmetry (space group *P6/mcc*) (e.g., Benito et al. [39]). The earlier works have shown that alkalis can be incorporated into the structural channels of cordierite. In this case, a charge of the Al/Si ratio in the framework balances according to the equation (K, Na)^+^ + Al^3+^ → Si^4+^, causing distortion of the hexagonal symmetry (e.g., [40,41,42,43]). Diffusion of potassium ions was documented during the dehydroxylation processes of muscovite and kaolinite and detected in metakaolinite layers above 900 °C [14]. The relation in Figure 5b shows a slightly larger lattice parameters *a* and *c* of indialites identified in ceramic samples in comparison with pure indialites designated as 1: Mg_2.00_Al_4.00_Si_5.00_O_18_ [4] and 2: Mg_2.00_Al_4.00_Si_5.00_O_18_ [44]. The similarly to the parameters of K-substituted indialites, designated as 3: (K_0.17_Mg_1.94_Fe_0.06_ Ca_0.04_)Al_4.25_Si_4.75_O_18_ [38] and 4: (K_0.25_Mg_1.75_)Al_4.25_Si_4.75_O_18_ [43] can be considered as the result of a possible migration and substitution of potassium ions into their structure.

**Figure 6:**
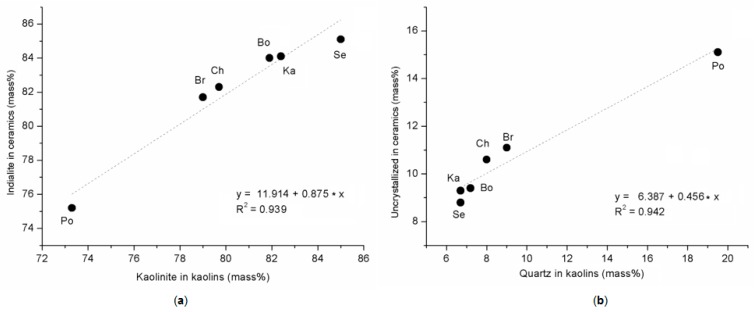
Content of mineral phases in ceramic samples in relation with minerals in kaolins: (**a**) indialite vs. amount of kaolinite and (**b**) non-crystalline phases vs. quartz.

Influence of kaolin on the quantity of crystallizing indialite/cordierite and quartz admixture in kaolin on the formulation of the non-crystalline phase is declared in Figure 6. The amounts of indialite/cordierite in the ceramic samples (Table 6, Figure 6a) correlate with the amount of kaolinite in kaolins (Table 5), as it was described by the linear regression function (Equation (10))

Indialite/cordierite (mass %) = 11.91 + 0.87 × kaolinite (mass %), R^2^ = 0.939 (10)

Similarly, the good relation between the amounts of non-crystalline phases in the ceramic samples (Table 6) and the amounts of quartz in kaolins (Table 5, Figure 6b) was described by the linear regression function (Equation (11))

Non-crystalline phases (mass %) = 6.39 + 0.46 × quartz (mass %), R^2^ = 0.942 (11)

**Figure 7:**
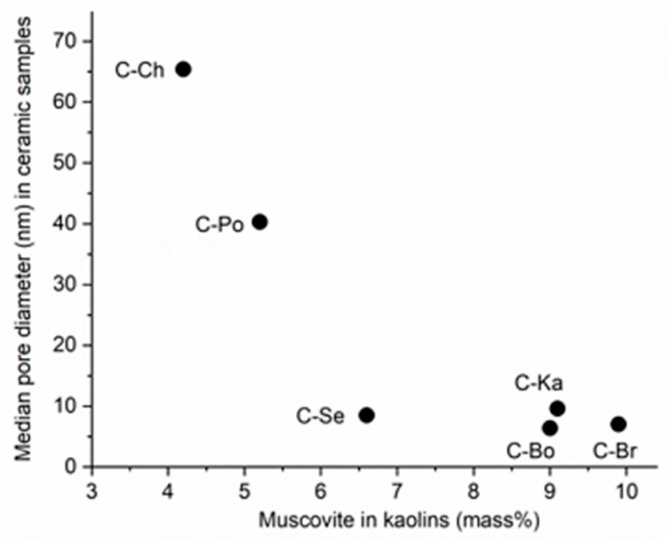
Median pore diameter in ceramic samples changing with content of muscovite in kaolins.

Cordierite samples C-Br, C-Ka, C-Bo, and C-Se prepared from kaolins containing muscovite in amount higher than 5 mass % (Table 5) were slightly more porous with smaller pores than cordierite samples C-Po and C-Ch (Table 7). Relation in Figure 7 shows the median pore diameter in ceramic samples decreasing with the content of muscovite in kaolins. Results from the porosimetry measurement of our ceramic samples are consistent with the literature observations about influence of muscovite on development of pores in ceramic body during the thermal transformations of kaolinite, e.g., [14,45,46]. 

## 5. Conclusions

Six raw kaolins containing kaolinite, quartz, and muscovite were sintered with vermiculite to the crystalline cordierite/indialite, enstatite and non-crystalline phases. The novel CQMA method first recalculates the elemental bulk analysis to the quantity of mineral phases in kaolins and the elemental bulk analysis of ceramic samples to the highest possible percentages of crystalline phases and the remaining elements to the non-crystalline phases. Potassium in the ceramic mixtures lowered the temperature difference from the melting to the crystallization. A possible migration of potassium into the crystal structure of indialite can be assumed according to the disordered unit cell parameters close to the K-doped indialites. Regression analysis showed high correlation between amounts of crystalline cordierite and kaolinite in kaolins as well as between quartz in kaolins and non-crystalline phases in the ceramics. Influence of muscovite on development of pores in ceramic body during the thermal transformations of kaolinite was confirmed.

## Figures and Tables

**Table 1:** ED-XRF analysis of kaolin samples.

Samples Hi ^1^	Bo 0.93	Se 1.08	Po 1.22	Ka 1.57	Br 1.59	Ch 1.30
Oxides (mass %)						
SiO_2_	49.97	49.65	56.25	49.71	50.83	50.45
TiO_2_	0.16	0.07	0.38	0.66	0.93	0.72
Al_2_O_3_	34.62	35.17	30.07	34.80	33.61	33.94
Fe_2_O_3_	1.10	0.86	0.72	0.72	0.67	0.72
CaO	0.15	0.12	0.25	0.09	0.01	0.06
MgO	0.22	0.21	0.24	0.21	0.17	0.27
Na_2_O	0.03	0.03	0.03	0.06	0.08	0.07
K_2_O	1.18	0.93	0.84	1.21	1.41	1.88
L.O.I. ^2^	12.21	12.26	11.15	12.26	12.01	11.63
Sum	99.66	99.30	99.94	99.72	99.72	99.75

^1^ Hinckley index [21]; ^2^ Loss on ignition after heating at 1000 °C for 2 h = H_2_O^+^.

**Table 2:** TG/DTA of kaolin samples.

Ranges:		1		2			3		4		Total Mass Loss	
Kaolin Sample	Mass (mg)	ΔT_1_ (°C)	Δ_m1_ (mg)	ΔT_2_ (°C)	Δ_m2_ (mg)	T_2min_ (°C)	ΔT_3_ (°C)	Δ_m3_ (mg)	ΔT_4_ (mg)	T_4max_ (°C)	Δm_TML_ (mg)	(%)
Bo	20.20	20–175	0.04	410–730	2.36	526	730–946	0.18	963–1031	988	2.58	12.8
Se	21.40	20–160	0.05	403–720	2.41	528	720–946	0.13	959–1041	990	2.59	12.1
Po	18.60	20–185	0.11	440–730	2.04	535	730–932	0.13	955–1041	993	2.28	12.3
Ka	20.90	20–200	0.06	450–725	2.32	535	750–946	0.16	961–1041	997	2.54	12.2
Br	19.90	20–170	0.01	430–720	2.19	533	730–931	0.13	972–1051	999	2.33	11.7
Ch	27.90	20–160	0.12	410–715	2.88	534	720–939	0.11	976–1047	997	3.11	11.1

**Table 3:** The ED-XRF analysis of ceramic samples.

Oxides	Samples					
(mass %)	C-Bo	C-Se	C-Po	C-Ka	C-Br	C-Ch
SiO_2_	52.88	52.82	56.01	52.71	53.29	52.95
TiO_2_	0.57	0.52	0.62	0.87	0.89	0.81
Al_2_O_3_	25.76	26.13	23.00	25.77	25.15	25.27
Fe_2_O_3_	4.68	4.53	4.48	4.45	3.99	4.44
MnO	0.06	0.06	0.05	0.05	0.05	0.05
CaO	0.81	0.75	0.86	0.73	0.70	0.70
MgO	13.25	13.24	13.19	13.28	13.22	13.26
Na_2_O	0.21	0.21	0.19	0.20	0.22	0.21
K_2_O	1.69	1.51	1.50	1.71	1.83	2.09
Sum	99.91	99.77	99.90	99.77	99.34	99.78

**Table 4:** TG/DTA of kaolin–vermiculite ceramic mixtures.

	Ranges:	1		2			3		4		5	Total Mass Loss	
Sample	Mass m (mg)	ΔT_1_ (°C)	Δm_1_ (mg)	ΔT_2_ (°C)	Δm_2_ (mg)	ΔT_5_ (°C)	ΔT_3_ (°C)	Δm_3_ (mg)	ΔT_4_ (°C)	T_4max_ (°C)	ΔT_5_ (°C)	Δm_TML_	
												(mg)	(%)
C-Bo	15.12	20–256	0.19	409–597	1.01	1084–1254	757–836	0.18	913–999	964	1084–1254	1.38	9.1
C-Se	15.00	20–226	0.18	416–610	1.01	1086–1255	755–843	0.19	906–1000	960	1086–1255	1.38	9.2
C-Po	15.11	20–233	0.19	407–631	0.93	1075–1252	753–838	0.18	905–1002	964	1075–1252	1.30	8.6
C-Ka	14.97	20–251	0.16	424–609	1.02	1080–1246	745–848	0.16	906–1017	960	1080–1246	1.34	8.9
C-Br	15.14	20–239	0.17	434–603	0.95	1102–1258	745–835	0.16	905–999	970	1102–1258	1.28	8.5
C-Ch	15.15	20–265	0.17	437–650	1.04	1104–1243	748–848	0.12	907–999	972	1104–1243	1.33	8.8

**Table 5:** Quantitative amounts of minerals in kaolin samples determined using CQMA method.

Mineral (mass %)	Samples					
	Bo	Se	Po	Ka	Br	Ch
Kaolinite	81.9	85.0	73.3	82.4	79.0	79.7
Muscovite	9.0	6.6	5.2	9.1	9.9	4.2
Quartz	7.2	6.7	19.5	6.7	9.0	6.0
Limonite	1.2	0.9	0.8	0.7	0.7	0.8
Rutile	0.1	0.1	0.3	0.6	0.9	0.7
Orthoclase	0.0	0.0	0.0	0.0	0.0	8.0
Sum	99.4	99.3	99.1	99.5	99.5	99.4

**Table 6:** Quantitative amounts of minerals in ceramic samples determined using CQMA method.

Mineral (mass %)	Samples					
	C-Bo	C-Se	C-Po	C-Ka	C-Br	C-Ch
Cordierite	84.0	85.1	75.2	84.1	81.7	82.3
Enstatite	6.2	5.8	8.9	6.5	6.8	6.7
Others ^(1)^	9.4	8.8	15.1	9.3	11.1	10.6
Sum	99.6	99.7	99.2	99.9	99.6	99.6

^(1)^ The calculated oxidic formulas of residual—non-crystalline phases based on 10SiO_2_: C-Bo: 0.29Na_2_O∙1.52K_2_O∙0.61TiO_2_∙10SiO_2_; C-Se: 0.31Na_2_O∙1.45K_2_O∙0.60TiO_2_∙10SiO_2_; C-Po: 0.15Na_2_O∙0.65K_2_O∙0.36TiO_2_∙10SiO_2_; C-Ka: 0.28Na_2_O∙1.72K_2_O∙0.96TiO_2_∙10SiO_2_; C-Br: 0.26Na_2_O∙1.41K_2_O∙0.82TiO_2_∙10SiO_2_; C-Ch: 0.27Na_2_O∙1.72K_2_O∙0.81TiO_2_∙10SiO_2_.

**Table 7:** Selected characteristics of ceramic samples from the porosimetry measurement.

	Samples					
	C-Bo	C-Se	C-Po	C-Ka	C-Br	C-Ch
Porosity (%)	3.52	3.49	1.77	4.60	3.00	1.24
MD (nm) ^(1)^	7.5	8.5	40.3	9.6	7.0	65.4

^(1)^ Median pore diameter (area).
